# Risk factors for aneurysm rupture among Kazakhs: findings from a national tertiary

**DOI:** 10.1186/s12883-022-02892-y

**Published:** 2022-09-20

**Authors:** Yerkin Medetov, Aisha Babi, Yerbol Makhambetov, Karashash Menlibayeva, Torekhan Bex, Assylbek Kaliyev, Serik Akshulakov

**Affiliations:** 1 Department of Vascular and Functional Neurosurgery, National Center for Neurosurgery, 34/1 Turan Avenue, Nur-Sultan, 010000 Kazakhstan; 2 Hospital Management Department, National Center for Neurosurgery, 34/1 Turan Avenue, Nur-Sultan, 010000 Kazakhstan

**Keywords:** Intracranial aneurysm, Aneurysm rupture, Kazakh population, Risk factors

## Abstract

**Background:**

Rupture of intracranial aneurysms (RIA) leads to subarachnoid hemorrhage (SAH) with severe consequences. Although risks for RIA are established, the results vary between ethnic groups and were never studied in Kazakhstan. This study aimed to establish the risk factors of RIA in the Kazakh population.

**Methods:**

Retrospective analysis of 762 patients with single IAs, who attended the neurosurgical center from 2008 until 2018, was conducted. Demographic characteristics, such as age, sex, smoking status, and hypertension were considered. Descriptive and bivariate analyses were performed. A multivariable logistic regression model was built to identify factors correlated with RIA.

**Results:**

The mean age of participants was 48.49 ± 0.44 years old. The majority (68.37%) of IAs have ruptured. Of the ruptured aneurysms, 43.76% were < 6 mm, and 38.39% were located on the anterior cerebral and anterior communicating arteries (ACA). Logistic regression model indicates younger age group (16–40 years), smoking, having stage 3 hypertension, smaller IA size and its location on ACA increase the odds of rupture.

**Conclusions:**

This study has revealed that younger, smoking patients with stage 3 arterial hypertension are at higher risk for RIA. Small aneurysms (< 6 mm) and location on ACA had increased odds of rupture, while larger aneurysms on internal carotid arteries had lower odds.

## Background

Intracranial saccular aneurysms develop when the wall of the cerebral artery becomes weak to resist hemodynamic pressure [1]. A RIA causes SAH associated with a high rate of mortality, with an in-hospital mortality rate of 18% [2], and permanent disability [3, 4]. SAH is attributed to 85% of IAs ruptures [5]. With such devastating outcomes, it is urgent to identify and prevent IAs from rupturing. However, many IAs remain stable throughout their lifetime, with one lifelong cohort study reporting one-third of rupture cases among the participants with IA [6]. Therefore, identifying markers differentiating stable IAs from those at risk of rupture would be essential to avoid unnecessary surgical interventions as well as avoiding neglect of the patients with SAH risks.

The prevalence of IAs was estimated to be 3.2% in the general population worldwide [7]. The population of Kazakhstan is around 18,700,000 people with around 67.5% of ethnic Kazakhs [8, 9]. About 500,000 people develop IAs, and the annual incidence of IAs is estimated to be 5.4 cases per 100,000 people [10, 11]. However, the true number of IAs in Kazakhstan remains unknown, as available statistics are limited to those who attended diagnostic clinics and hospitals.

Age, sex, arterial hypertension, smoking, history of SAH, aneurysm size, and location are all known risk factors for RIA [12–14]. The average age of the population with IA varies according to the previous studies [15–19]. A large multinational study has found that the average age of patients with IA is 50.4 years old [17]. Country-specific findings varied, the average age of IA diagnosis is 53.2 years old in Finland [18], 52 years old in the Netherlands [16], and 60–63 years old in Japan [15, 19]. According to the large cohort study, the IAs occurrence ratio discrepancy between sexes in the adult population was 3:1 female to male [20]. It was found that the size of aneurysms in 90% of cases is less than 10 mm in diameter and anterior cerebral circulation accounts for 90% of IA locations [21]. More than 20–29% of the population with IAs have multiple aneurysms [13, 22]. In addition, ethnic factors [12, 14] and irregular IAs shape [15, 23, 24] play a role in the risk of rupture.

Significant differences in RIA risk factors were found among different study population groups. Specifically, several Japanese [15, 25], Finnish [6, 18] and Australian [26] studies have demonstrated higher risks of RIAs < 7 mm in size than what has been previously reported in several international studies [20, 27]. This study aims to define the risk factors associated with RIA among Kazakhs, as no such work has been done prior.

## Methods

### Study population

The study included 762 patients with single saccular IA of the cerebral vessels treated in the Department of Vascular and Functional Neurosurgery, National Center for Neurosurgery during the 10 year period from 2008 to 2018. Patients of Kazakh ethnicity and with saccular IA were included in the study. Ethnicity was defined through self-reported information. Data were derived from the patients’ electronic and paper medical records.

### Variables

Age, sex, smoking, arterial hypertension, size, and location of an aneurysm were analyzed. Arterial hypertension was classified into three groups according to WHO classification [28]. Information on arterial hypertension was derived from the unified Hospital Information System, self-reported information, and daily examination results.

### Diagnosis, location, and sizes of IAs

IAs were diagnosed based on the CT, MRA, and CA data. SAH was diagnosed based on the CT scans. Vascular neurosurgeons reviewed the images. In cases of patients who had clinical symptoms of SAH, but had non-definitive CT scans, a lumbar puncture was taken for laboratory diagnosis to verify the diagnosis.

In this study, the location of an aneurysm was grouped into ICA, PCA, MCA, and ACA. Anterior communicating artery and anterior cerebral arteries were abbreviated as ACA. Posterior communicating, posterior cerebral, vertebral, and basilar arteries were abbreviated as PCA. The maximal diameter of an aneurysm was designated as the size of an aneurysm. IAs were grouped into four groups according to Yasargil's classification [29].

### Statistical analysis

Data were analyzed using STATA 16 (Stata Corp, 2019). Descriptive analysis included reporting mean values with standard deviation and frequencies, where appropriate. The normal distribution of continuous data was checked using histograms and scatter plots. Association between continuous and categorical variables was tested with Student’s t-test, Wilcoxon signed-rank test, and one-way analysis of variance (ANOVA), where appropriate. For categorical variables, the Chi-square test or Fisher’s exact tests were used. Logistic regression model was built using a backward stepwise approach to identify factors associated with an increase in aneurysm rupture risk. Assumptions for logistic regression were tested with link test (_hat *p*-value < 0.001, _hatsq *p*-value = 0.934). Pearson goodness-of-fit test was used to estimate the fit of the model (*p*-value = 0.1858). *P*-value < 0.05 was considered statistically significant.

## Results

Seven hundred sixty-two patients with single aneurysm diagnosis data were reviewed (Table 1). Among them, 68% had ruptured aneurysms and approximately 32% had unruptured aneurysms. The mean age of the patients was 48.49 ± 0.44 years with the youngest being 16 years old and the oldest 81 years old. RIA occurred at a significantly higher proportion (79.66%) among the youngest age group of 16–40 years old. Approximately 60% of the participants were women and 40% were men. However, rupture occurred in men more frequently than in women. A quarter of the patients were smokers and almost 84% of smokers had ruptured aneurysms. Just under 30% of the patients had no arterial hypertension, and about 43% had stage 3 hypertension. The proportion of aneurysm rupture was significantly higher (80.66%) among those with stage 3 hypertension. The rest were diagnosed with stage 1 (4.72%) and stage 2 (22.97%) hypertension. The average size of the aneurysms was 9.81 ± 0.29 mm. Unruptured aneurysms were significantly larger than those that did rupture (13.41 ± 0.67 vs 8.15 ± 0.27). The majority of the aneurysms fell into the medium size category (44.09%); however, small-sized aneurysms had the highest proportion of rupture (78.62%). Most of the aneurysms (38.71%) were located on the ICA, but ACA had the highest proportion (87.82%) of ruptured aneurysms.

**Table 1:** Demographic and anthropometric characteristics of the study participants

Variable	Ruptured aneurysms, *n* = 521 (68.37%)	Unruptured aneurysms, *n* = 241 (31.63%)	*p*-value	All, *N* = 762 (100%)
Age (years), mean ± SD	47.13 ± 0.52	51.42 ± 0.77	< 0.001	48.49 ± 0.44
Age group (years), n (%)				
16–40	141 (79.66%)	36 (20.34%)	< 0.001	177 (23.23%)
41–49	137 (69.19%)	61 (30.81%)		198 (25.98%)
50–56	118 (69.41%)	52 (30.59%)		170 (22.31%)
57–81	125 (57.60%)	92 (42.40%)		217 (28.48%)
Sex				
Female	286 (63.41%)	165 (36.59%)	< 0.001	451 (59.19%)
Male	235 (75.56%)	76 (24.44%)		311 (40.81%)
Smoking				
No	360 (63.16%)	210 (36.84%)	< 0.001	570 (74.80%)
Yes	161 (83.85%)	31 (16.15%)		192 (25.20%)
Arterial hypertension				
Normal	129 (58.64%)	91 (41.36%)		220 (28.87%)
Stage 1 hypertension	18 (50%)	18 (50%)	< 0.001	36 (4.72%)
Stage 2 hypertension	107 (61.14%)	68 (38.86%)		175 (22.97%)
Stage 3 hypertension	267 (80.66%)	64 (19.34%)		331 (43.44%)
Aneurysm size (mm), mean ± SD	8.15 ± 0.27	13.41 ± 0.67	< 0.001	9.81 ± 0.29
Aneurysm size group, n (%)				
Small, < 6 mm	228 (78.62%)	62 (21.38%)	< 0.001	290 (38.06%)
Medium, ≥ 6 mm or < 15	235 (69.94%)	101 (30.06%)		336 (44.09%)
Large, ≥ 15 mm or < 25 mm	40 (50.63%)	39 (49.37%)		79 (10.37%)
Giant, ≥ 25 mm	18 (31.58%)	39 (68.42%)		57 (7.48%)
Location				
ICA	149 (50.51%)	146 (49.49%)	< 0.001	295 (38.71%)
ACA	200 (87.82%)	28 (12.28%)		228 (29.92%)
MCA	151 (75.50%)	49 (24.50%)		200 (26.25%)
PCA	21 (53.85%)	18 (46.15%)		39 (5.12%)

**Fig. 1:**
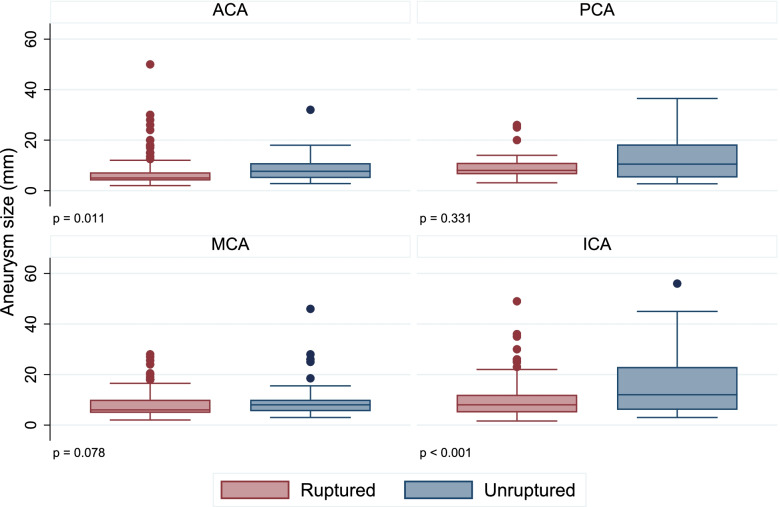
Correlation of ruptured and unruptured aneurysm size with aneurysm location. *p*-value was calculated with Wilcoxon signed-rank test. ACA – anterior cerebral and anterior communicating arteries, ICA – internal carotid artery, MCA – middle cerebral artery, PCA – posterior communicating, posterior cerebral, vertebral, and basilar arteries

The correlation between RIAs size and the location of the IAs was found in ACA and ICA (Figure 1). As can be seen from the Figure the distribution of sizes was right-skewed and significantly smaller among RIAs in ACA and ICA. No such correlation was found at MCA and PCA.

Multivariable logistic regression was built to determine factors associated with aneurysm rupture. All variables that showed statistical significance and had a biological basis were considered for the model. Our model included categorical age, sex of the participants, aneurysm size, aneurysm location, arterial hypertension, and smoking as factors influencing the odds of rupture (Table 2). Compared to the youngest age group (16–40 years), other age groups had decreasing odds of rupture with the lowest likelihood (0.07 times) in the oldest age group, when adjusted for other variables. Men had 17% higher odds of rupture, when compared to women and adjusted for other variables. An increase in size decreased the rupture likelihood when compared to the small aneurysms with the smallest odds of 0.22 in giant aneurysms when adjusting to other variables. IA’s location on ACA compared to ICA increased the adjusted odds of rupture 5.43 times. Patients with stage 3 hypertension had a 12.4 times higher likelihood of aneurysm rupture when compared to patients with normal arterial blood pressure and adjusting for other variables. The odds of smokers increased by 86% when compared to non-smokers and adjusted for other variables.

**Table 2:** Bivariable crude odds ratio and multivariable adjusted odds ratio of factors associated with aneurysm rupture

Variable	COR (95% CI)	*p*-value	AOR (95% CI)	*p*-value
Age (years)				
16–40	Reference	< 0.001	Reference	< 0.001
41–49	0.57 (0.36 ± 0.92)		0.23 (0.12 ± 0.44)	
50–56	0.58 (0.35 ± 0.95)		0.16 (0.08 ± 0.32)	
57–81	0.35 (0.22 ± 0.55)		0.07 (0.03 ± 0.14)	
Sex				
Female	Reference	< 0.001	Reference	0.5
Male	1.78 (1.29 ± 2.46)		1.17 (0.74 ± 1.86)	
Aneurysm size				
Small, < 6 mm	Reference	< 0.001	Reference	< 0.001
Medium, ≥ 6 mm or < 15	0.63 (0.44 ± 0.91)		0.71 (0.47 ± 1.09)	
Large, ≥ 15 mm or < 25 mm	0.28 (0.17 ± 0.47)		0.48 (0.26 ± 0.9)	
Giant, ≥ 25 mm	0.13 (0.07 ± 0.23)		0.22 (0.1 ± 0.47)	
Location				
ICA	Reference	< 0.001	Reference	< 0.001
ACA	7 (4.43 ± 11.05)		5.43 (3.16 ± 9.33)	
MCA	3.02 (2.03 ± 4.48)		2.5 (1.57 ± 4.01)	
PCA	1.14 (0.59 ± 2.23)		0.95 (0.44 ± 2.06)	
Arterial hypertension				
Normal	Reference	< 0.001	Reference	< 0.001
Stage 1 hypertension	0.71 (0.35 ± 1.43)		1.39 (0.52 ± 3.72)	
Stage 2 hypertension	1.11 (0.74 ± 1.66)		2.9 (1.6 ± 5.24)	
Stage 3 hypertension	2.94 (2.01 ± 4.31)		12.4 (6.71 ± 22.91)	
Smoking				
No	Reference	< 0.001	Reference	0.03
Yes	3.03 (1.99 ± 4.61)		1.86 (0.77 ± 3.31)	

## Discussion

This study aimed to identify risk factors associated with RIA in the Kazakh population. To our knowledge, this is the first study to analyze differentiating factors between those with ruptured and unruptured IAs. The participants of the study were patients who underwent treatment in the NCN located in the capital of Kazakhstan, Nur-Sultan, which admits patients from all over the country.

From the results of the logistical model, such factors as younger age, active smoking status, stage 3 hypertension, location of the aneurysm on ACA, and smaller aneurysm size (< 6 mm) increased the odds of aneurysm rupture. Although studies have shown an association of female sex with reduced risk of aneurysm rupture [30], in the model of this study the factor of sex becomes insignificant when adjusted for other variables. In an unadjusted analysis, men had 1.78 times higher odds of IA rupture, but when adjusted for other variables the odds fell to 1.17. Unadjusted smoking had increased the odds of rupture 3 times, but adjustment for other variables lowered it to 1.86 times, indicating a possible confounding effect. Among our participants, 87.5% of smokers were men.

In line with findings from other studies [30–32], the youngest age group in our sample had higher odds of RIA. There are several possible explanations for such observation. As was noted [31] in a previous study, this might be due to the slower blood flow rate, as well as due to the calcification of arterial walls among older patients. Meanwhile, Zhang and colleagues [32] found an association between the younger age of the participants and morphological features that are more likely to lead to the rupture, such as the presence of daughter and irregular domes, larger flow angle, and other features. Unfortunately, such factors were not available for analysis in this study.

Hypertension was another factor that had the largest effect on the odds of rupture. Stage 3 hypertension increased the odds of RIA by 12 times when compared to the patients with normal blood pressure. Similar results to a varying degree were found in other studies [33–35].

Multivariable logistic regression showed that aneurysms located on ACA had 5.43 times increase in odds of rupture compared to ICA. Different studies have found different sites to be more prone to rupture. For example, the ARETA study found an association of ACA/Acom location with RIA [35], while the PHASES study had shown a higher correlation of RIA with PCA and PCoA [12]. In this study sample, the majority (38.71%) of the aneurysms were located on ICA, but only 50.51% of them had ruptured. Meanwhile, among the 29.92% of aneurysms that were located on ACA 87.82% have ruptured. Overall, aneurysms located on ACA accounted for 38.39% of all ruptured IAs in our sample.

Interestingly, smaller aneurysms in this study had a higher likelihood of rupture, while large and giant IAs had a lower probability of rupture. The correlation of rupture with the size of the aneurysm was tested in different locations of the aneurysm, and RIAs on ACA were statistically significantly smaller than unruptured IAs in the same location. Similarly, the same correlation with size was found on ICA. PCA and MCA did not have a significant difference in the sizes of ruptured and unruptured aneurysms. This result confirms findings that suggest anterior communicating artery aneurysms have a higher probability of rupture at a smaller size [36].

There also could be a genetic factor influencing the prevalence of RIA on ACA among our sample. As was found in a previous study, 13 SNPs are associated with the risk of development and rupture of aneurysms in the Kazakh population [37] Other factors, such as morphological features of IAs, were found to be good predictors of IAs rupture [38]. However, no correlation was found between morphological features that predispose to rupture with the size of IA [39]. Therefore, a subset of small aneurysms can be at heightened risk for rupture, which could be the case in our sample.

A study conducted in Finland has shown that 68% of RIA were smaller than 10 mm [18]. The majority of the aneurysms among the population they studied were located on MCA and ACoA. The results differ from those found in a pooled analysis, which indicated location on PCoA as well as aneurysms size ≥ 20 mm as risk factors for North American and European countries [12]. Finland and Japan were the only exceptions. A Japanese study has shown 64.6% of the small IAs (< 6 mm) and 73.9% of medium IAs (≥ 6–15 mm) have ruptured with the majority of the IAs located on ACA [40], which is similar to findings in our study.

Although the PHASES score [12] developed to estimate the risk of RIA suggests minimal risks of rupture for those younger than 70 and with smaller IA (< 10 mm), these recommendations are contrary to the findings of the studies conducted in Japan and Finland. Our study has also confirmed the necessity for a more thorough examination of IAs without the assumption that smaller IA would not rupture. As was noted by Zanaty et al. there is an increasing pool of evidence suggesting a high risk of rupture for smaller aneurysms. The study also emphasized the importance of establishing specific indicators that identify the instability of the small aneurysms at risk of rupture [39]. Population-specific studies are important for establishing more accurate risk factors for the populations, as the findings suggest that increased attention to smaller IAs could decrease SAH incidences among Kazakhs.

### Limitations

This study has its limitations, one of which is the nature of a retrospective study. Such important variables as diabetes status, alcohol consumption, and severity of smoking, presence of atherosclerosis, family medical history of the patients were not studied. In addition, the data were collected from one neurosurgical center, which mainly performs elective surgeries. It was not possible to include patients who have passed away suddenly from SAH. This could distort the size average of the RIA reported. Convenience sampling of the participants does not allow the authors to find causation, only association, and does not allow us to extrapolate our findings to the whole population.

## Conclusions

This is the first study to examine factors associated with RIA among the Kazakh population. This study has identified younger age, smoking status, stage 3 hypertension, size < 6 mm, and location on ACA as risk factors for RIA among the Kazakhs. Although the larger size of IAs is a major risk factor in many North American and European studies, the Kazakh population coincides with findings from the Finnish and Japanese cohorts, where smaller IAs were at higher risk of rupture. This study confirms the need for a more thorough examination of IA on such aspects as morphology regardless of the size, and the need to pay more sensibility to ethnic differences in risk factors, especially in ethnically diverse countries. Future prospective cohort studies should be conducted to better understand the etymology of IA.

## Data Availability

All the data collected and used for this study is available for public viewing via the link https://zenodo.org/record/5637874.

## Abbreviations

ACA: Anterior cerebral and anterior communicating arteries
AR: Aspect ratio
CA: Cerebral angiography
CT: Computed tomography
IA: Intracranial aneurysm
ICA: Internal carotid artery
IRB: Institutional Review Board
MCA: Middle cerebral artery
MRA: Magnetic resonance angiography
NCN: National Center for Neurosurgery
PCA: Posterior communicating, posterior cerebral, vertebral and basilar arteries
RIA: Rupture of intracranial aneurysms
SAH: Subarachnoid haemorrhage
SNP: Single nucleotide polymorphism
SBP: Systolic blood pressure
WHO: World Health Organization

## References

[1] Schievink WI (1997). Intracranial aneurysms. N Engl J Med.

[2] Lantigua H, Ortega-Gutierrez S, Schmidt JM, Lee K, Badjatia N, Agarwal S (2015). Subarachnoid hemorrhage: who dies, and why?. Crit Care Lond Engl.

[3] Petridis AK, Kamp MA, Cornelius JF, Beez T, Beseoglu K, Turowski B (2017). Aneurysmal Subarachnoid Hemorrhage. Dtsch Arzteblatt Int.

[4] D’Souza S (2015). Aneurysmal Subarachnoid Hemorrhage. J Neurosurg Anesthesiol.

[5] Tawk RG, Hasan TF, D’Souza CE, Peel JB, Freeman WD (2021). Diagnosis and Treatment of Unruptured Intracranial Aneurysms and Aneurysmal Subarachnoid Hemorrhage. Mayo Clin Proc.

[6] Korja M, Lehto H, Juvela S (2014). Lifelong rupture risk of intracranial aneurysms depends on risk factors: a prospective Finnish cohort study. Stroke.

[7] Vlak MH, Algra A, Brandenburg R (2011). Rinkel GJ Prevalence of unruptured intracranial aneurysms, with emphasis on sex, age, comorbidity, country, and time period: a systematic review and meta-analysis. Lancet Neurol.

[8] Lillis J. Joanna Lillis on the Secret World of Kazakhstan - Voices On Cental Asia. I.B. Tauris; 2018.

[9] The World Bank. Kazakhstan | Data [Internet]. [cited 2021 Sep 12]. Available from: https://data.worldbank.org/country/KZ

[10] Akshulakov SК, Medetov EZ, Jamantayeva BD, Kaliyev AB, Zholdybayeva YB, Aitkulova AM. Некоторые клинико-эпидемиологические аспекты разорвавшихся интракраниальных аневризм у пациентов казахской национальности [Some clinico-epidemiologycal aspects of the ruptured intracranial aneurysms in patients of kazakh nationality]. J Neurosurg Neurol Kazakhstan. 2016.

[11] Medetov EZ, Makhambetov YT, Zholdybayeva YB, Aitkulova AM, Kaliyev AB, Berdibayeva DT, Zholmagambetova M. Анализ прогностических факторов при интракраниальных аневризмах в казахской популяции [Analysis of prognostic factors for intracranial aneurysms in the Kazakh population]. J Neurosurg Neurol Kazakhstan. 2018;3(52):3–10.

[12] Greving JP, Wermer MJ, Brown RD, Morita A, Juvela S, Yonekura M (2014). Development of the PHASES score for prediction of risk of rupture of intracranial aneurysms: a pooled analysis of six prospective cohort studies. Lancet Neurol.

[13] Murayama Y, Takao H, Ishibashi T, Saguchi T, Ebara M, Yuki I (2016). Risk Analysis of Unruptured Intracranial Aneurysms: Prospective 10-Year Cohort Study. Stroke.

[14] Wermer MJ, van der Schaaf IC, Algra A, Rinkel GJ (2007). Risk of rupture of unruptured intracranial aneurysms in relation to patient and aneurysm characteristics: an updated meta-analysis. Stroke..

[15] Morita A, Kirino T, Hashi K, Aoki N, Fukuhara S, Hashimoto N (2012). The natural course of unruptured cerebral aneurysms in a Japanese cohort. N Engl J Med.

[16] Wermer MJ, van der Schaaf IC, Velthuis BK, Majoie CB, Albrecht KW, Rinkel GJ (2006). Yield of short-term follow-up CT/MR angiography for small aneurysms detected at screening. Stroke.

[17] Kassell NF, Torner JC, Haley EC, Jane JA, Adams HP, Kongable GL (1990). The International Cooperative Study on the Timing of Aneurysm Surgery. Part 1: Overall management results. J Neurosurg.

[18] Korja M, Kivisaari R, Rezai Jahromi B, Lehto H (2017). Size and location of ruptured intracranial aneurysms: consecutive series of 1993 hospital-admitted patients. J Neurosurg.

[19] Sonobe M, Yamazaki T, Yonekura M, Kikuchi H (2010). Small unruptured intracranial aneurysm verification study: SUAVe study. Japan. Stroke.

[20] International Study of Unruptured Intracranial Aneurysms Investigators. Unruptured intracranial aneurysms--risk of rupture and risks of surgical intervention. N Engl J Med. 1998;339(24):1725–33. 10.1056/NEJM199812103392401 9867550

[21] Steiner T, Juvela S, Unterberg A, Jung C, Forsting M, Rinkel G (2013). European Stroke Organization guidelines for the management of intracranial aneurysms and subarachnoid haemorrhage. Cerebrovasc Dis Basel Switz.

[22] Huttunen T, von und zu Fraunberg M, Frösen J, Lehecka M, Tromp G, Helin K (2010). Saccular intracranial aneurysm disease: distribution of site, size, and age suggests different etiologies for aneurysm formation and rupture in 316 familial and 1454 sporadic eastern Finnish patients. Neurosurgery.

[23] Kleinloog R, De Mul N, Verweij BH, Post JA, Rinkel GJ, Ruigrok YM (2018). Risk Factors for Intracranial Aneurysm Rupture: A Systematic Review. Neurosurgery..

[24] Lindgren AE, Koivisto T, Björkman J, von und zu Fraunberg M, Helin K, Jääskeläinen JE (2016). Irregular Shape of Intracranial Aneurysm Indicates Rupture Risk Irrespective of Size in a Population-Based Cohort. Stroke.

[25] Ikawa F, Hidaka T, Yoshiyama M, Ohba H, Matsuda S, Ozono I (2019). Characteristics of Cerebral Aneurysms in Japan. Neurol Med Chir (Tokyo)..

[26] Froelich JJ, Neilson S, Peters-Wilke J, Dubey A, Thani N, Erasmus A (2016). Size and Location of Ruptured Intracranial Aneurysms: A 5-Year Clinical Survey. World Neurosurg..

[27] Wiebers DO, Whisnant JP, Huston J, Meissner I, Brown RD, Piepgras DG, et al. Unruptured intracranial aneurysms: natural history, clinical outcome, and risks of surgical and endovascular treatment. Lancet. 2003;362(9378):103–10. 10.1016/s0140-6736(03)13860-3 12867109

[28] World Health Organization (1978). Arterial Hypertension.

[29] Yaşargil MG. Microneurosurgery. Thieme; 1984. p. 410.

[30] Can A, Castro VM, Ozdemir YH, Dagen S, Yu S, Dligach D (2017). Association of intracranial aneurysm rupture with smoking duration, intensity, and cessation. Neurology.

[31] Liberato AC, Xu J, Montes D, Heit JJ, Barnaure I, Maza NM (2020). Multivariable analysis on factors associated with aneurysm rupture in patients with multiple intracranial aneurysms. Emerg Radiol.

[32] Zhang J, Lai PMR, Can A, Mukundan S, Castro VM, Dligach D (2021). Tobacco use and age are associated with different morphologic features of anterior communicating artery aneurysms. Sci Rep.

[33] Bonita R (1986). Cigarette smoking, hypertension and the risk of subarachnoid hemorrhage: a population-based case-control study. Stroke.

[34] Jaja BNR, Saposnik G, Lingsma HF, Macdonald E, Thorpe KE, Mamdani M, et al. Development and validation of outcome prediction models for aneurysmal subarachnoid haemorrhage: the SAHIT multinational cohort study. BMJ. 2018;360:j5745. 10.1136/bmj.j5745 29348138

[35] Pierot L, Barbe C, Ferré JC, Cognard C, Soize S, White P (2020). Patient and aneurysm factors associated with aneurysm rupture in the population of the ARETA study. J Neuroradiol.

[36] Rinaldo L, Nesvick CL, Rabinstein AA, Lanzino G (2020). Differences in Size Between Unruptured and Ruptured Saccular Intracranial Aneurysms by Location. World Neurosurg..

[37] Zholdybayeva EV, Medetov YZ, Aitkulova AM, Makhambetov YT, Akshulakov SK, Kaliyev AB (2018). Genetic Risk Factors for Intracranial Aneurysm in the Kazakh Population. J Mol Neurosci MN..

[38] Texakalidis P, Sweid A, Mouchtouris N, Peterson EC, Sioka C, Rangel-Castilla L (2019). Aneurysm Formation, Growth, and Rupture: The Biology and Physics of Cerebral Aneurysms. World Neurosurg.

[39] Zanaty M, Daou B, Chalouhi N, Starke RM, Jabbour P, Hasan D (2016). Evidence That a Subset of Aneurysms Less Than 7 mm Warrant Treatment. J Am Heart Assoc.

[40] Orz Y, Kobayashi S, Osawa M, Tanaka Y (1997). Aneurysm size: a prognostic factor for rupture. Br J Neurosurg..

