# Correction: Risk factors for aneurysm rupture among Kazakhs: findings from a national tertiary hospital

**DOI:** 10.1186/s12883-022-02901-0

**Published:** 2022-09-29

**Authors:** Yerkin Medetov, Aisha Babi, Yerbol Makhambetov, Karashash Menlibayeva, Torekhan Bex, Assylbek Kaliyev, Serik Akshulakov

**Affiliations:** 1 Department of Vascular and Functional Neurosurgery, National Center for Neurosurgery, 34/1 Turan Avenue, Nur-Sultan, Kazakhstan 010000; 2 Hospital Management Department, National Center for Neurosurgery, 34/1 Turan Avenue, Nur-Sultan, Kazakhstan 010000

**Correction: BMC Neurol 22, 357 (2022)**

**https://doi.org/10.1186/s12883-022-02892-y**

Following publication of the original article [1], the authors reported an error in the article title. The word “hospital” is missing. The correct title should be:

“Risk factors for aneurysm rupture among Kazakhs: findings from a national tertiary hospital”

The original article [1] has been updated.

## References

[1] Medetov Y, Babi A, Makhambetov Y (2022). Risk factors for aneurysm rupture among Kazakhs: findings from a national tertiary hospital. BMC Neurol.

